# Supplementary material for: Adult Attachment and Emotion Regulation Flexibility in Romantic Relationships
Source: Behav Sci (Basel). 2024 Aug 27;14(9):758. doi: 10.3390/bs14090758 (PMC11428407; doi:10.3390/bs14090758)
Supplement: Supplementary file 1 [file behavsci-14-00758-s001.zip › behavsci-3071598-supplementary.pdf]

**Table S1**

*Demographic Information of Studies*

| | Study 1 | | Study 2 Baseline | | Study 2 Baseline + ESM | |
|---|---|---|---|---|---|---|
| Characteristic | n | % | n | % | n | % |
| *Gender* | | | | | | |
| Female | 133 | 76% | 244 | 85% | 104 | 84% |
| Male | 41 | 24% | 40 | 14% | 20 | 16% |
| Other | 0 | 0% | 3 | 1% | 0 | 0% |
| *Ethnicity* | | | | | | |
| White/Caucasian | 151 | 87% | 249 | 87% | 111 | 90% |
| Black/African-American | 1 | 1% | 1 | 0% | 0 | 0% |
| Hispanic/Latino | 0 | 0% | 2 | 1% | 1 | 1% |
| Asian | 6 | 3% | 13 | 5% | 3 | 2% |
| Middle-Eastern | 5 | 3% | 7 | 2% | 2 | 2% |
| Mixed | 8 | 5% | 10 | 3% | 4 | 3% |
| Other ethnicity | 3 | 2% | 5 | 2% | 3 | 2% |
| *Marriage status* | | | | | | |
| Married | 12 | 7% | 12 | 4% | 7 | 6% |
| Unmarried | 162 | 93% | 275 | 96% | 117 | 94% |
| *Cohabitation* | | | | | | |
| Yes | 66 | 38% | 79 | 28% | 32 | 26% |
| No | 108 | 62% | 208 | 72% | 92 | 74% |
| *Children* | | | | | | |
| Yes | 8 | 5% | 11 | 4% | 4 | 3% |
| No | 166 | 95% | 276 | 96% | 120 | 97% |
| *Education level* | | | | | | |
| No formal qualification | 1 | 1% | 0 | 0% | 0 | 0% |
| Secondary school/High schoo | 94 | 54% | 171 | 60% | 78 | 63% |
| Some college, no degree | 20 | 11% | 28 | 10% | 12 | 10% |
| Undergraduate degree (BA/BSc/other) | 42 | 24% | 62 | 22% | 19 | 15% |
| Graduate degree (MA/MSc/MPhil/other) | 16 | 9% | 24 | 8% | 14 | 11% |
| Doctorate degree (PhD/MD/other) | 1 | 1% | 2 | 1% | 1 | 1% |

**Table S2**

*Between-Person Correlations of Variables In Study 1 and Study 2 Baseline*

| | Variable | 1 | 2 | 3 | 4 | 5 | 6 | 7 | 8 | 9 | 10 | 11 | 12 |
|---|---|---|---|---|---|---|---|---|---|---|---|---|---|
| 1 | Inter-vs-intrapersonal ER | _ | 0.84*** | -0.85*** | -0.13 | -0.39*** | -0.05 | -0.04 | 0.19* | -0.27*** | 0.01 | -0.10 | 0.29*** |
| 2 | Interpersonal ER | 0.89*** | _ | -0.42*** | -0.11 | -0.42*** | -0.07 | -0.07 | 0.20 | -0.30*** | 0.00 | -0.05 | 0.29*** |
| 3 | Intrapersonal ER | -0.89*** | -0.57*** | _ | 0.10 | 0.24** | 0.02 | 0.00 | -0.13 | 0.15* | -0.03 | 0.11 | -0.20* |
| 4 | Attachment anxiety | -0.05 | -0.03 | 0.05 | _ | 0.41*** | -0.20** | -0.21** | -0.42*** | -0.25** | -0.03 | 0.26*** | 0.02 |
| 5 | Attachment avoidance | -0.42*** | -0.44*** | 0.31*** | 0.30*** | _ | 0.06 | 0.07 | -0.68*** | 0.06 | -0.07 | 0.26*** | -0.12 |
| 6 | Age (years) | 0.02 | 0.07 | 0.04 | -0.02 | -0.01 | _ | 0.80*** | -0.10 | 0.10 | 0.07 | -0.22** | -0.17* |
| 7 | Relationship length (months) | 0.05 | 0.08 | -0.01 | -0.18** | -0.11 | 0.65*** | _ | -0.13 | 0.06 | 0.00 | -0.17* | -0.08 |
| 8 | Relationship quality | 0.08 | 0.13* | -0.01 | -0.44*** | -0.41*** | -0.06 | 0.03 | _ | 0.02 | 0.05 | -0.25** | 0.14 |
| 9 | Neuroticism | -0.08 | -0.08 | 0.06 | -0.39*** | -0.07 | -0.02 | 0.03 | -0.03 | _ | 0.05 | -0.11 | -0.32*** |
| 10 | Extraversion | 0.14* | 0.11 | -0.13* | -0.11 | -0.11 | -0.08 | -0.09 | 0.01 | 0.12* | _ | 0.13 | 0.19* |
| 11 | COVID | -0.11 | -0.12* | 0.07 | 0.21*** | 0.16** | -0.04 | -0.12* | -0.13* | -0.14* | 0.04 | _ | 0.11 |
| 12 | Female | 0.15* | 0.10 | -0.17** | 0.11 | 0.05 | -0.07 | 0.01 | -0.03 | -0.22*** | -0.07 | 0.04 | _ |

*Note*. Study 1 correlations are presented on the top diagonal; Study 2 Baseline correlations are presented on the bottom diagonal.

**Table S3**

*Analysis of Attachment Influencing Emotion Regulation Across Studies*

| | Study 1 Baseline | | | | Study 2 Baseline | | | | Study 2 Baseline + ESM | | | | |
|---|---|---|---|---|---|---|---|---|---|---|---|---|---|
| Variable | *b* | *SE* | *t* | *p* | *b* | *SE* | *t* | *p* | *b* | *SE* | *DF* | *t* | *p* |
| | | | | | | Outcome: Interpersonal ER | | | | | | | |
| (Intercept) | **3.19** | **0.16** | **19.89** | **<.001** | **3.39** | **0.16** | **21.44** | **<.001** | **2.07** | **0.35** | **2492** | **5.96** | **<.001** |
| Attachment anxiety | -0.06 | 0.10 | -0.61 | 0.542 | 0.07 | 0.07 | 1.06 | 0.290 | 0.12 | 0.16 | 113 | 0.73 | 0.469 |
| Attachment avoidance | **-0.61** | **0.12** | **-5.04** | **<.001** | **-0.62** | **0.08** | **-7.72** | **<.001** | **-0.66** | **0.20** | **113** | **-3.35** | **0.001** |
| Attachment anxiety: Attachment avoidance | -0.05 | 0.09 | -0.56 | 0.577 | 0.09 | 0.06 | 1.44 | 0.151 | 0.15 | 0.15 | 113 | 1.02 | 0.311 |
| Relationship length | 0.00 | 0.00 | -0.71 | 0.477 | 0.00 | 0.00 | 0.77 | 0.445 | **-0.01** | **0.00** | **113** | **-2.03** | **0.045** |
| Female | **0.51** | **0.19** | **2.73** | **0.007** | 0.27 | 0.17 | 1.58 | 0.116 | 0.28 | 0.36 | 113 | 0.79 | 0.432 |
| Others | | | | | -0.22 | 0.58 | -0.37 | 0.711 | | | | | |
| Relationship quality | -0.25 | 0.13 | -1.93 | 0.056 | -0.05 | 0.09 | -0.54 | 0.591 | 0.04 | 0.23 | 113 | 0.17 | 0.869 |
| Neuroticism | **-0.15** | **0.05** | **-2.78** | **0.006** | -0.07 | 0.05 | -1.50 | 0.134 | 0.19 | 0.10 | 113 | 1.89 | 0.061 |
| Extraversion | -0.03 | 0.04 | -0.80 | 0.426 | 0.07 | 0.04 | 1.69 | 0.092 | 0.06 | 0.09 | 113 | 0.65 | 0.518 |
| COVID | 0.00 | 0.00 | -0.13 | 0.897 | 0.00 | 0.00 | -1.56 | 0.119 | 0.01 | 0.01 | 113 | 1.55 | 0.123 |
| Stressfulness of event | | | | | | | | | **0.19** | **0.04** | **2492** | **5.05** | **<.001** |
| Partner availability | | | | | | | | | **3.98** | **0.23** | **2492** | **17.33** | **<.001** |
| Others availability | | | | | | | | | 0.09 | 0.14 | 2492 | 0.62 | 0.533 |
| Time | | | | | | | | | 0.00 | 0.00 | 2492 | -0.50 | 0.617 |
| | | | | | | Outcome: Intrapersonal ER | | | | | | | |
| (Intercept) | **3.88** | **0.19** | **20.65** | **<.001** | **3.80** | **0.16** | **23.13** | **<.001** | **7.79** | **0.34** | **2473** | **22.91** | **<.001** |
| Attachment anxiety | 0.07 | 0.12 | 0.59 | 0.559 | 0.06 | 0.07 | 0.78 | 0.438 | 0.12 | 0.16 | 113 | 0.73 | 0.465 |
| Attachment avoidance | **0.33** | **0.14** | **2.30** | **0.023** | **0.48** | **0.08** | **5.75** | **<.001** | 0.17 | 0.20 | 113 | 0.83 | 0.408 |
| Attachment anxiety: Attachment avoidance | -0.03 | 0.11 | -0.29 | 0.774 | -0.06 | 0.07 | -0.97 | 0.331 | **-0.31** | **0.15** | **113** | **-2.14** | **0.035** |
| Relationship length | 0.00 | 0.00 | -0.09 | 0.927 | 0.00 | 0.00 | 0.46 | 0.649 | 0.00 | 0.00 | 113 | 0.14 | 0.890 |
| Female | -0.42 | 0.22 | -1.91 | 0.058 | **-0.47** | **0.18** | **-2.64** | **0.009** | -0.21 | 0.37 | 113 | -0.57 | 0.568 |
| Others | | | | | 0.65 | 0.60 | 1.07 | 0.284 | | | | | |
| Relationship quality | 0.14 | 0.15 | 0.93 | 0.353 | **0.22** | **0.09** | **2.38** | **0.018** | 0.05 | 0.24 | 113 | 0.21 | 0.834 |
| Neuroticism | 0.08 | 0.06 | 1.35 | 0.179 | 0.07 | 0.05 | 1.49 | 0.138 | -0.09 | 0.10 | 113 | -0.89 | 0.373 |
| Extraversion | 0.00 | 0.05 | -0.02 | 0.986 | **-0.08** | **0.04** | **-1.97** | **0.050** | -0.09 | 0.09 | 113 | -1.05 | 0.295 |
| COVID | 0.00 | 0.00 | 1.08 | 0.283 | 0.00 | 0.00 | 0.83 | 0.407 | -0.01 | 0.01 | 113 | -1.17 | 0.246 |
| Stressfulness of event | | | | | | | | | 0.04 | 0.03 | 2473 | 1.28 | 0.202 |
| Partner availability | | | | | | | | | **-1.89** | **0.18** | **2473** | **-10.72** | **<.001** |
| Others availability | | | | | | | | | **-1.01** | **0.15** | **2473** | **-6.97** | **<.001** |
| Time | | | | | | | | | **0.01** | **0.00** | **2473** | **2.00** | **0.046** |

*Note*. All continuous predictors are centered. The significant values are presented in bold.

**Table S4**

*Between-Person and Within-Person Correlations of Variables in Study 2 Baseline + ESM*

Between-person correlations

| Variable | 1 | 2 | 3 | 4 | 5 | 6 | 7 | 8 | 9 | 10 | 11 | 12 | 13 | 14 | 15 | 16 |
|---|---|---|---|---|---|---|---|---|---|---|---|---|---|---|---|---|
| *Baseline measures* | | | | | | | | | | | | | | | | |
| 1.Inter-vs-intrapersonal ER | | | | | | | | | | | | | | | | |
| 2.Interpersonal ER | 0.92 | | | | | | | | | | | | | | | |
| 3.Intrapersonal ER | -0.92 | -0.68 | | | | | | | | | | | | | | |
| 4.Attachment anxiety | -0.08 | -0.06 | 0.09 | | | | | | | | | | | | | |
| 5.Attachment avoidance | -0.52 | -0.52 | 0.42 | 0.30 | | | | | | | | | | | | |
| 6.Relationship length (months) | 0.08 | 0.15 | 0.00 | -0.21 | -0.17 | | | | | | | | | | | |
| 7.Relationhsip quality | 0.12 | 0.16 | -0.06 | -0.36 | -0.48 | 0.00 | | | | | | | | | | |
| 8.Neuroticism | -0.06 | -0.09 | 0.02 | -0.37 | -0.11 | 0.12 | 0.02 | | | | | | | | | |
| 9.Extraversion | 0.15 | 0.10 | -0.19 | -0.18 | -0.07 | -0.13 | 0.02 | 0.15 | | | | | | | | |
| 10.COVID | -0.24 | -0.27 | 0.16 | 0.11 | 0.26 | -0.15 | -0.08 | -0.09 | -0.03 | | | | | | | |
| 11.female | 0.15 | 0.13 | -0.13 | 0.07 | 0.01 | 0.00 | 0.01 | -0.27 | -0.09 | -0.04 | | | | | | |
| *ESM measures* | | | | | | | | | | | | | | | | |
| 12.Inter-vs-intrapersonal ER | 0.31 | 0.22 | -0.35 | -0.09 | -0.29 | -0.11 | 0.19 | 0.06 | 0.10 | 0.05 | 0.03 | | | | | |
| 13.Interpersonal ER | 0.29 | 0.26 | -0.27 | -0.02 | -0.35 | -0.15 | 0.22 | 0.04 | 0.06 | 0.02 | 0.02 | 0.87 | | | | |
| 14.Intrapersonal ER | -0.23 | -0.10 | 0.31 | 0.15 | 0.12 | 0.04 | -0.10 | -0.06 | -0.10 | -0.07 | -0.03 | -0.82 | -0.43 | | | |
| 15.Stressfulness of event | -0.11 | -0.05 | 0.15 | 0.06 | 0.01 | -0.14 | 0.08 | -0.16 | -0.05 | 0.00 | 0.19 | 0.13 | 0.26 | 0.06 | | |
| 16.Partner availability | 0.17 | 0.14 | -0.18 | -0.02 | -0.22 | -0.05 | 0.16 | -0.22 | -0.04 | -0.01 | 0.07 | 0.55 | 0.56 | -0.36 | 0.13 | |
| 17.Others availability | 0.11 | 0.08 | -0.12 | 0.02 | 0.01 | 0.13 | -0.14 | 0.13 | 0.07 | -0.15 | -0.06 | 0.13 | 0.04 | -0.18 | 0.00 | -0.18 |

Within-person correlations

| Variable | 1 | 2 | 3 | 4 | 5 | 6 |
|---|---|---|---|---|---|---|
| 1.Inter-vs-intrapersonal ER | | | | | | |
| 2.Interpersonal ER | 0.88 | | | | | |
| 3.Intrapersonal ER | -0.81 | -0.44 | | | | |
| 4.Stressfulness of event | 0.10 | 0.19 | 0.05 | | | |
| 5.Partner availability | 0.46 | 0.49 | -0.26 | 0.06 | | |
| 6.Others availability | 0.07 | 0.00 | -0.14 | 0.00 | -0.12 | |
| 7.Time | -0.01 | 0.01 | 0.03 | 0.01 | 0.05 | -0.02 |

- $p < .05$
- $p < .01$
- $p < .001$

**Table S5**

*Comparing Variable Scores Between Studies*

| | | Study 1 versus Study 2 Baseline | | | | | Study 2 Baseline versus Study 2 Baseline + ESM | | | | |
|---|---|---|---|---|---|---|---|---|---|---|---|
| | | t-test | | | Non parametric Wilcoxon test | | t-test | | | Non parametric Wilcoxon test | |
| | Variable | t | df | p | w | p | t | df | p | w | p |
| 1 | Inter-vs-intrapersonal ER | 1.37 | 372.57 | .172 | 26662 | .217 | 1.03 | 447.93 | .302 | 33636 | .363 |
| 2 | Interpersonal ER | 0.76 | 365.38 | .447 | 25859 | .503 | 0.12 | 468.9 | .902 | 32455 | .846 |
| 3 | Intrapersonal ER | -1.59 | 353.06 | .114 | 22858 | .115 | -0.24 | 462.62 | .807 | 32211 | .967 |
| 4 | Attachment anxiety | 0.78 | 416.64 | .439 | 25437 | .736 | -1.19 | 498.54 | .236 | 30836 | .429 |
| 5 | **Attachment avoidance** | **2.12** | **352.92** | **.035** | **28532** | **.010** | 0.53 | 484.80 | .596 | 33281 | .492 |
| 6 | **Age (years)** | **-2.56** | **265.84** | **.011** | **20667** | **.002** | 0.90 | 415.3 | .370 | 31788 | .882 |
| 7 | Relationship length (months) | -1.94 | 202.78 | .053 | 22990 | .531 | 1.22 | 412.87 | .225 | 32416 | .550 |
| 8 | **Relationship quality** | **2.08** | **363.08** | **.038** | **28317** | **.016** | 1.30 | 508.02 | .200 | 32877 | .658 |
| 9 | Neuroticism | 0.93 | 346.85 | .353 | 26480 | .274 | 0.60 | 476.4 | .547 | 33362 | .459 |
| 10 | Extraversion | 1.20 | 320.75 | .231 | 26197 | .374 | -0.80 | 473.47 | .426 | 30876 | .442 |
| 11 | **COVID** | **2.86** | **374.85** | **.005** | **29134** | **.003** | 0.60 | 472.64 | .547 | 32995 | .607 |
| 12 | **Female** | **2.23** | **317.53** | **.027** | **27112** | **.021** | -0.74 | 464.61 | .463 | 31364 | .459 |

*Note*. The significant values are presented in bold.

**Table S6**

*Analysis of Attachment Influencing Within-Strategy Variability*

| | Interpersonal ER | | | | Intrapersonal ER | | | |
|---|---|---|---|---|---|---|---|---|
| Variable | *b* | *SE* | *t* | *p* | *b* | *SE* | *t* | *p* |
| (Intercept) | **2.96** | **0.38** | **7.87** | **<.001** | **1.96** | **0.35** | **5.68** | **<.001** |
| Attachment anxiety | -0.13 | 0.11 | -1.23 | 0.222 | **-0.23** | **0.10** | **-2.32** | **0.022** |
| Attachment avoidance | 0.04 | 0.14 | 0.31 | 0.756 | 0.07 | 0.13 | 0.51 | 0.613 |
| Attachment anxiety: Attachment avoidance | 0.04 | 0.10 | 0.36 | 0.718 | **0.24** | **0.09** | **2.55** | **0.012** |
| Relationship length | -0.00 | 0.00 | -0.75 | 0.453 | -0.00 | 0.00 | -1.55 | 0.125 |
| Relationship quality | 0.11 | 0.16 | 0.69 | 0.490 | 0.02 | 0.15 | 0.12 | 0.907 |
| Neuroticism | 0.01 | 0.07 | 0.13 | 0.899 | -0.04 | 0.06 | -0.67 | 0.504 |
| COVID | 0.00 | 0.00 | 0.26 | 0.792 | 0.01 | 0.00 | 1.89 | 0.061 |
| Participant level mean stressfulness of events | -0.04 | 0.06 | -0.75 | 0.453 | -0.00 | 0.05 | -0.08 | 0.934 |
| Participant level mean partner availability | **1.00** | **0.42** | **2.40** | **0.018** | **0.81** | **0.38** | **2.11** | **0.037** |
| Participant level mean others availability | 0.17 | 0.44 | 0.38 | 0.706 | 0.50 | 0.40 | 1.23 | 0.220 |

*Note.* The significant values are presented in bold.

**Table S7**

*Moderation Effect of Attachment on the Association Between Partner Availability and Inter-Vs-Intrapersonal ER*

| Variable | *b* | *SE* | *DF* | *t* | *p* |
|---|---|---|---|---|---|
| | Outcome: Interpersonal ER | | | | |
| (Intercept) | **2.05** | **0.35** | **2494** | **5.81** | **<.001** |
| Attachment anxiety | 0.33 | 0.19 | 113 | 1.70 | 0.092 |
| Attachment avoidance | **-0.77** | **0.24** | **113** | **-3.23** | **0.002** |
| Partner availability | **4.04** | **0.23** | **2494** | **17.41** | **<.001** |
| Attachment anxiety: Attachment avoidance | 0.23 | 0.18 | 113 | 1.28 | 0.204 |
| Attachment anxiety: Partner availability | **-0.50** | **0.23** | **2494** | **-2.17** | **0.030** |
| Attachment avoidance: Partner availability | 0.23 | 0.29 | 2494 | 0.78 | 0.436 |
| Attachment anxiety: Attachment avoidance: Partner availability | -0.24 | 0.26 | 2494 | -0.96 | 0.335 |
| Relationship length | **-0.01** | **0.00** | **113** | **-2.03** | **0.045** |
| Female | 0.28 | 0.35 | 113 | 0.91 | 0.367 |
| Relationship quality | 0.04 | 0.23 | 113 | 0.15 | 0.884 |
| Neuroticism | 0.18 | 0.10 | 113 | 1.81 | 0.074 |
| Extraversion | 0.06 | 0.09 | 113 | 0.69 | 0.491 |
| COVID | 0.01 | 0.01 | 113 | 1.54 | 0.125 |
| Stressfulness of event | **0.19** | **0.04** | **2494** | **5.14** | **<.001** |
| Others availability | 0.09 | 0.14 | 2494 | 0.66 | 0.507 |
| Time | -0.00 | 0.00 | 2494 | -0.46 | 0.648 |
| | Outcome: Intrapersonal ER | | | | |
| (Intercept) | **7.80** | **0.34** | **2485** | **22.98** | **<.001** |
| Attachment anxiety | 0.11 | 0.16 | 113 | 0.66 | 0.508 |
| Attachment avoidance | 0.17 | 0.20 | 113 | 0.82 | 0.413 |
| Partner availability | **-1.92** | **0.18** | **2485** | **-10.56** | **<.001** |
| Attachment anxiety: Attachment avoidance | **-0.32** | **0.15** | **113** | **-2.18** | **0.031** |
| Attachment anxiety: Partner availability | 0.10 | 0.18 | 2485 | 0.56 | 0.573 |
| Attachment avoidance: Partner availability | 0.02 | 0.23 | 2485 | 0.07 | 0.943 |
| Attachment anxiety: Attachment avoidance: Partner availability | 0.14 | 0.21 | 2485 | 0.68 | 0.494 |
| Relationship length | 0.00 | 0.00 | 113 | 0.13 | 0.899 |
| Female | -0.22 | 0.37 | 113 | -0.59 | 0.558 |
| Relationship quality | 0.05 | 0.23 | 113 | 0.21 | 0.831 |
| Neuroticism | -0.09 | 0.10 | 113 | -0.89 | 0.373 |
| Extraversion | -0.10 | 0.09 | 113 | -1.07 | 0.287 |
| COVID | -0.01 | 0.01 | 113 | -1.16 | 0.250 |
| Stressfulness of event | 0.04 | 0.03 | 2485 | 1.18 | 0.239 |
| Others availability | **-1.02** | **0.14** | **2485** | **-7.05** | **<.001** |
| Time | **0.01** | **0.00** | **2485** | **2.10** | **0.035** |

*Note.* The significant values are presented in bold.

**Table S8**

*Exploratory Correlations between Study Variables and Other Related Variables Measured During Data Collection of Study 2*

| Variable | 2 | 3 | 4 | 5 | 6 | 7 | 8 | 9 | 10 |
|---|---|---|---|---|---|---|---|---|---|
| *Baseline measures* | | | | | | | | | |
| 1. Attachment anxiety | 0.30*** | -0.06 | -0.05 | -0.06 | 0.04 | < 0.001 | -0.07 | 0.21* | 0.15 |
| 2. Attachment avoidance | – | -0.53*** | -0.34*** | 0.15 | -0.46*** | -0.38*** | 0.01 | 0.21* | 0.21* |
| 3. Interpersonal ER | | – | 0.45*** | -0.36*** | 0.66*** | 0.46*** | -0.06 | -0.09 | -0.11 |
| 4. Interpersonal ER efficacy | | | – | -0.20* | 0.29** | 0.35*** | -0.04 | -0.10 | -0.13 |
| 5. Intrapersonal ER efficacy | | | | – | -0.31*** | -0.27** | 0.07 | 0.09 | 0.10 |
| 6. IRQ tendency | | | | | – | 0.66*** | -0.01 | 0.03 | 0.03 |
| 7. IRQ efficacy | | | | | | – | -0.14 | -0.04 | -0.09 |
| *ESM measures* | | | | | | | | | |
| 8. Compliance rate | | | | | | | – | -0.10 | 0.34*** |
| 9. Rate of reported stressful events | | | | | | | | – | 0.89*** |
| 10. Sum of reported stressful events | | | | | | | | | – |

*Note*. IRQ is Interpersonal Regulation Questionnaire [74]. For a complete list of variables, see https://osf.io/et5a2

**Table S9**

*Deviations of the Studies From Pre-Registration*

| Pre-registration link | Study ID | Pre-registered | Deviation of the study from pre-registration | Explanation |
|---|---|---|---|---|
| https://osf.io/m2t9g | 1 | Analysis: Correlation | Analysis: Correlation and Linear Regression | Regression analysis was added in order to have a more comprehensive analysis, including both attachment orientations, their interaction effect, and control variables. Notably, the results of correlation and regression analyses did not significantly differ from one another in the significance and direction of the effect. |
| https://osf.io/et5a2 | 2 | Inclusion criteria: currently involved in a romantic relationship longer than 6 months | Inclusion criteria: currently involved in a romantic relationship longer than 3 months | In spite of indicating inclusion criteria during participant recruitment, few participants completed ESM part while having relationships less than 6 months. Since ESM data collection is valuable, we decided to make this criteria less conservative and reduced it to 3 months. Several studies have considered 3 months enough for similar analysis; thus, our decision is well-informed. |
| https://osf.io/et5a2 | 2 | Hypothesis: H1.1. and H1.2 | Beyond Hypotheses H1.1 and H1.2 that were included in the pre-registration, we included Hypotheses (H2.1, H2.2, H3.1, H3.2, H3.3). Furthermore, we did not investigate the hypotheses H3, H4, and H5 of the pre-registration in the current manuscript. | After reading literature, we decided to also look at other aspects of ER variability and flexibility, thus new hypotheses.<br>Given the expanded focus on ER variability and flexibility, we chose not to investigate the pre-registered hypotheses H3, H4, and H5 in the current manuscript. The decision to exclude these hypotheses was driven by a desire to concentrate on the analysis of interpersonal versus intrapersonal ER. Specifically, the exploration of emotional experiences and specific ER strategies (such as rumination) fell outside the scope of this manuscript. |
